# Validation of an Automated Method for the Isolation and Purification of Fat-Soluble Vitamins and Cholesterol for Chromatographic Analysis

**DOI:** 10.1093/jaoacint/qsaf011

**Published:** 2025-02-17

**Authors:** Marleen van Aardt, Andrew R Komarek, Michael Roche, Elise Ivarsen

**Affiliations:** ANKOM Technology, 2052 O’Neil Rd, Macedon, NY 14502, USA; ANKOM Technology, 2052 O’Neil Rd, Macedon, NY 14502, USA; Columbia Laboratories, Inc., 12423 NE Whitaker Way, Portland, OR 97230, USA; Eurofins Vitamin Testing, Ladelundvej 85, DK-6600 Vejen, Denmark

## Abstract

**Background:**

The isolation and purification of vitamins A, E, D, and cholesterol from food and feed test materials, for quantitation, is currently a time-consuming and labor-intensive process. It includes separate steps for saponification, extraction, purification, and solvent evaporation. A new instrument (FLEX) was developed that improves and automates all steps involved, and which uses solid-phase extraction (SPE). This study validates the FLEX automated method.

**Objectives:**

The objective of this study was to validate the automated method by recovery of standards, analysis of reference materials, comparison against proficiency test materials, and comparison against manual reference methods.

**Methods:**

The FLEX instrument automatically adds reagents, mixes, and heats to saponify test materials, filters the digestate, extracts with SPE, and evaporates solvent.

**Results:**

The accuracy of the automated FLEX instrument method was confirmed by the agreement with National Institute of Standards and Technology (NIST) reference materials for retinol, α-tocopherol, cholecalciferol, ergocalciferol, and cholesterol. Accuracy was also compared against manual reference methods on 11 different food types that ranged from 4–100% fat, 0–75% protein, and 0–85% carbohydrate. The automated and manual methods were highly correlated with no bias or distortion over the range of test materials. Precision was similar to the manual methods for retinol recovery but improved for α-tocopherol and cholecalciferol analysis. The accuracy of the automated method also was confirmed for feed analysis. Eleven different animal feeds were analyzed in the FLEX instrument and results were highly correlated with Association of American Feed Control Officials proficiency test results.

**Conclusion:**

The automated method accurately and efficiently performed the multiple analytical steps necessary for the isolation and purification of the analytes in preparation for chromatographic analysis.

**Highlights:**

The automated method was compared against industry standard methods and yielded equivalent results and improved precision. SPE technology was optimized to efficiently elute non-polar analytes, while retaining protein and other medium-polar analytes.

The nutritional composition of food and feed ingredients is fundamental to the evaluation of the food or feed as a nutrient source for humans and animals. Nutritional analysis is a multibillion-dollar industry with the global market estimated to double from 2018 to 2026 and the vitamin segment dominating the market (1). As consumers become more aware of the value of food nutrition and safety, the demand for nutritious food and beverages has increased significantly. As a result, the demand for analysis associated with populating nutrition facts labels has increased accordingly.

The analysis of vitamins A, E, D, and other micro-ingredients involves procedures for the isolation and purification of the test portion prior to quantifying chromatographically. These procedures are time-consuming and labor-intensive. They generally include manual steps for alkaline saponification (if hydrolysis of the ester is required), filtration or centrifugation, isolation by liquid–liquid or solid-phase extraction (SPE), and the evaporation of solvent to yield the concentrated extract.

Many manual methods exist but each have limitations. Analysts are repeatedly handling hazardous chemicals and are at risk of injury due to shaking separatory funnels, and losses in analyte recovery and errors are seen as a result of unwanted emulsions, oxygen contamination, and manual transfers of test solutions. One of the major limitations of most manual methods is liquid–liquid extraction. Not only does it require a great deal of glassware, but it also often results in the formation of emulsions, which take a long time to form a clear separation between solvent and aqueous layers and can result in loss of analyte. Many foods contain emulsifiers, whether natural or added, which exacerbates this issue. SPE is an alternative extraction method, which is being used more frequently for this application, and which is not confounded by emulsion issues (2–5). Due to the manual operations involved in the isolation of fat-soluble vitamins and the limitations of liquid–liquid extraction, the FLEX instrument was developed to automate all the steps involved in the isolation of these analytes while employing SPE technology.

The objective of this study was to validate the automated method. Accuracy and precision were evaluated for a range of food and feed test materials. Four analytes (retinol, α-tocopherol, cholecalciferol, and cholesterol) were analyzed at concentration ranges commonly found in food, pet food, and animal feed (retinol: 0.6–37 600 µg/100 g; α-tocopherol: 1.3–1570 mg/100 g; cholecalciferol: 0.7–2800 µg/100 g; and cholesterol: 13–466 mg/100 g). Test materials included 4 Certified reference materials (CRMs), 11 food and pet food test materials, and 11 animal feed test materials (Association of American Feed Control Officials (AAFCO) 202121–202132). Two certified testing laboratories (Columbia Laboratories, Portland, OR, USA and Eurofins Steins Laboratory, Vejen, Denmark) and one research and development laboratory (ANKOM Technology, Macedon, NY, USA) collaborated in this study. Each collaborator had FLEX instruments on site and was able to perform both automated and manual methods. Quantification of analytes was performed on various analytical instruments (LC–Ultraviolet (UV), LC–Diode Array Detector (DAD), LC–MS/MS, and GC–Flame Ionization Detector (FID) as specified by the methods of the testing location. This study validates the first automated method for removing matrix interferences prior to quantitative analysis of vitamins A, E, D, and cholesterol from food and feed.

## Experimental

### Chemicals, Materials, and Equipment

This study is a collaboration between three laboratories, with each laboratory using a FLEX instrument on site at the time of validation. The manual method used to compare against the automated method included European standards (EN) EN12823–1 (retinol), EN12822 (α-tocopherol), and EN 12821 (cholecalciferol). All other equipment and reagents are specific to the testing location and are listed below.

### ANKOM^FLEX^ Analyte Extractor

The FLEX instrument consists of three distinct compartments: a digestion oven, an in-line SPE section for separation, and a recovery and evaporation chamber (ANKOM Technology). A touch pad and computer provide ease of use and access to controls and logs. The digestion vessels fit into the digestion oven and consist of glass and Teflon parts that sandwich filter disks. SPE cartridges contain silica-based sorbent material. Glass recovery vessels fit into the recovery chamber where solvent is evaporated.

### Analytical Instruments

- *Test location 1.*—
  1. *Retinol (all-trans and 13-cis) and α-tocopherol*.—The HPLC–DAD/Fluorescence Detector (FLD) system consisted of a Series 1200 binary gradient pump, ChemStation software, and Series 1200 UV/Diode Array and Fluorescence detectors (Agilent). The chromatographic separation was performed on a YMC-Pack PVA-Sil, 5 µm column, 4.6 × 250 mm (YMC America). All-*trans* and 13-*cis* retinol were quantified at 325 nm against an external calibration curve in a linear range of 0.25–76 µg/mL. α-Tocopherol was quantified at an excitation wavelength of 230 nm and an emission wavelength of 310 nm, also against an external calibration curve, in a linear range of 1–400 µg/mL.
- *Test location 2.—*
  1. *Retinol (all-trans and 13-cis).*—The HPLC–DAD system consisted of Ultimate 3000, Chromeleon software and an Ultimate 3000 RS UV/Diode Array detector (Thermo Scientific). The chromatographic separation was performed on a Hypersil GOLD silica, 5 µm column, 2.1 × 100 mm (Thermo Scientific). All-*trans* and 13-*cis* retinol were quantified at 325nm against an external calibration curve in a linear range of 0.75–2.5 µg/mL.
  2. *α-Tocopherol.*—The HPLC–FLD system consisted of an Ultimate 3000 RS pump, Chromeleon software, and an Ultimate 3000 Fluorescence detector (Thermo Scientific). The chromatographic separation was performed on a Luna silica, 3 µm column, 100Å, 4.6 × 250 mm (Phenomenex). α-Tocopherol was quantified at an excitation wavelength of 290 nm and an emission wavelength of 327 nm, against an external calibration curve, in a linear range of 2.5–100 µg/mL.
  3. *Cholecalciferol.*—The HPLC–DAD system consisted of a Vanquish Flex 2D Ultra-high pressure liquid chromatography (UHPLC) pump, Chromeleon software, and a Vanquish UV/Diode Array detector (Thermo Scientific). The chromatographic separation was performed on two columns: (1) semi-preparative: Accucore 2.6 µm column, 4.6 × 100 mm and (2) analytical: Accucore 2.6 µm column, 3 × 100 mm (Thermo Scientific). Cholecalciferol was quantified at 325 nm in a linear range of 2–120 µg/mL. Ergocalciferol was used as internal standard.
- *Test location 3.—*
  1. *Cholecalciferol and ergocalciferol*.—The UHPLC–MS/MS system consisted of a Nexera UHPLC system (Shimadzu), Analyst software, and a 5500 series Triple Quadrupole Mass Spectrometer detector (Sciex). The chromatographic separation was performed on a Luna Omega Polar C18, 100 × 2.1 mm, 1.6 μm (Phenomenex). Vitamin D_2_ and D_3_ were quantified in a linear range of 1–500 ng/mL.
  2. *Cholesterol.*—The GC–FID system consisted of a 6890 series Gas Chromatograph, ChemStation software, and a flame-ionization detector (Agilent). The chromatographic separation was performed on a ZB-5HT 30 m × 0.25 mm x 0.10 µm column (Phenomenex). Cholesterol was quantified using an internal standard in a linear range of 1–1000 µg/mL.

### Reference and Test Materials

- *CRMs*.—NIST 1869, Infant/Adult Nutritional Formula II; NIST 1546a, Meat Homogenate; and NIST 3290, Dry Cat Food.
- *Food (for method comparison)*.—Infant formula (Allomin, Semper), dry pet food (Royal Canin), wet pet food (Cora), sunflower seed margarine (Tartine & Caisson), cod liver oil, infant cereal (Blemil Riso 3), infant growth milk, drinking yogurt (Sainsbury’s), Fish Feed Std 600 (Ewos).
- *Food (for spike study)*.—Infant formula (Enfamil Milk-Based with Iron Infant Formula Powder, 0–12 Months, Mead Johnson); salmon jerky (Wild Alaskan Smoked Sockeye Salmon Jerky, Trident Seafoods).
- *Animal feed*.—AAFCO Equine Feed 202121, Beef Feed 202123, Pig Feed 202124, Dry Dog Feed 202125, Poultry Feed 202126, Rabbit Feed 202127, Sheep Feed 202128, Goat Mineral 202199, Hog Finisher 202130, Beef Feed 202131, and Poultry Feed 202132.

### Reagents

- *Standards*.—All-*trans* retinol (≥95% and ≥98%), α-tocopherol (≥95.0% and ≥95.5%), ergocalciferol (≥98%, 40 000 000 IU/g), cholecalciferol (≥98%, 40 000 000 IU/g), cholesterol (≥99%), 5-α-cholestane (≥97%), vitamin D_3_-[^2^H_6_] in ethanol, and N-(trimethylsilyl)imidazole (TMSI; >98%, ACS grade).
- *Chemicals*.—*n*-Hexane (HPLC grade), hexane (ACS grade), *n*-heptane (HPLC grade), 2-propanol (HPLC grade), 95% ethanol (ACS grade), methanol (HPLC grade), pyridine (≥99%). potassium hydroxide (flakes and pellets), butylated hydroxytoluene (BHT), and pyrogallol (99%).

### Preparation of Reagents

- 2% (w/v) Pyrogallol in 95% ethanol.
- Potassium hydroxide (KOH) solution (12.7N, or 50%, w/w).
- *n*-Hexane with 0.05 mg/mL BHT.
- All-*trans* retinol standard solutions in *n*-heptane (25 µg/mL).
- Cholesterol standard solution in *n*-hexane (500 µg/mL).
- 5-α-Cholestane standard solution in *n*-hexane (500 µg/mL).
- Cholecalciferol (vitamin D_3_) in methanol (500 µg/mL) for HPLC–DAD.
- Cholecalciferol [^2^H_6_] (vitamin D_3_) internal standard (5 ppm) for UHPLC–MS/MS.

### Automated Method

Solution reservoirs contained (1) 2% pyrogallol in 95% ethanol, (2) 12.7 N KOH, (3) Deionized water (DI) water, and (4) *n*-hexane containing 0.05 mg/mL BHT. Digestion vessels were assembled to include vitamin filters. Test portions were weighed directly into the vessels. The automated method was selected on the instrument touchscreen and started. The method performed the following steps:

- Purge the digestion vessels, SPE columns, and recovery vessels with nitrogen, to provide an inert environment to minimize oxidation degradation.
- Fill each digestion vessel with saponification solutions (25 mL ethanol, 10 mL KOH).
- Mix and saponify for 45 min at 75°C. Sealed digestion vessels are heated with directed hot air. Temperature and pressure are controlled and logged.
- Add 23 mL water after saponification to aid in rapid cool-down and to adjust the water:ethanol ratio to 1:1.
- Cool the saponified solution to 60°C.
- Filter and transfer the saponified solution onto SPE columns.
- Wash and elute the analyte with hexane.
- Evaporate solvent from the recovery vessels to yield a residue. Nitrogen is used to agitate and spread the eluent in the recovery vessels into a thin film while exhausting solvent vapor and maintaining an inert environment.
- The residue contained isolated vitamins and unsaponifiable components, such as cholesterol, ready for reconstitution and quantification.

### Quantification

Quantification of analytes was specific to the testing locations listed below.

- *Test location 1.*—
  1. *Retinol and α-tocopherol (HPLC–DAD/FLD)*.—The isolate was reconstituted quantitatively with *n*-heptane (2–10 mL) to ensure that dilutions were within the calibration range of the analyte. An aliquot of the reconstitute was filtered through a 0.22 µm membrane PTFE filter, transferred to an HPLC vial, and analyzed.
- *Test location 2.—*
  1. *Retinol (HPLC–DAD).*—An aliquot of the eluent was filtered through a 0.45 µm membrane PTFE filter, transferred to an HPLC vial, and analyzed.
  2. *α-Tocopherol (HPLC–FLD)*.—An aliquot of the reconstitute was filtered through a 0.45 µm membrane PTFE filter, diluted if necessary to ensure that dilutions were within the calibration range of the analyte and transferred to an HPLC vial, and analyzed.
  3. *Vitamin D_3_ and D_2_ (HPLC–DAD)*.—An aliquot of the eluent was evaporated under nitrogen and reconstituted quantitatively with 20% diethyl ether in *n*-heptane. The reconstitute was transferred to a 10 mL tube and the solvent evaporated. The residue was reconstituted in methanol, and thereafter filtered through a 0.45 µm membrane PTFE filter and transferred to an HPLC vial for 2D HPLC.
- *Test location 3.—*
  1. *Vitamin D_3_ and D_2_ (UPLC–MS/MS).*—The residue was reconstituted with methanol to a final volume of 1 mL, centrifuged at 17 000 revolutions per minute for 10 min. The supernatant was then transferred to a vial and analyzed.
  2. *Cholesterol (GC–FID).*—The residue was quantitatively reconstituted with hexane (approximately10–12 mL), aliquoted for cholesterol analysis, and evaporated to dryness under nitrogen with gentle heat (<40°C). The aliquot was then reconstituted with 1 mL pyridine and 500 µL TMSI, transferred to a vial, and analyzed.

## Results

### Reference Materials

Accuracy was investigated by comparing recoveries from the automated method using the FLEX instrument to the published values by NIST for three CRMs (NIST 1869, Infant formula; NIST 3290, Dry Cat Food; and NIST 1546a, Meat Homogenate). All analytes tested fell within the 95% confidence interval, as reported by NIST (Table 1). The manual methods used by NIST collaborating laboratories were as follows: vitamin A (AOAC Methods **992.04**, **992.06**, **2001.13**, **2002.06**, **2011.07**, **2012.10**/International Organization for Standardization (ISO) 20633:2015, EN 12823-1:2009, and ISO 12080–1:2009); vitamin E (AOAC Methods **992.03**, **2012.09**, **2012.10**/ISO 20633.2015, and EN 12822:2014); vitamin D (AOAC Methods **979.24**, **980.26**, **982.29**, **992.26**, **995.05**, **2002.05**, **2011.11**, **2012.11**, **2016.05**, and EN ISO 12521.2009); and cholesterol (AOAC Methods **933.08**, **970.50**, **970.51**, **994.10**, and American Oil Chemists Society (AOCS) Ce 12–16). Most fat-soluble vitamin methods employ ethanolic saponification, while separation includes both liquid–liquid extraction and SPE. The automated method compared well with these industry standard methods. Table 1 also lists the precision of the automated method. RSD was consistently <5% across all types of analytes, concentration of analyte, and food matrix tested.

**Table 1.** Method accuracy and precision against reference materials

Analyte	NIST CRM	NIST reported value			Automated method					
		Mean	Units	U_95%_^a^	Mean	Units	SD	Recovery, %	RSD, %	*n*
Retinol^b^	1869, Infant Formula	19.27	µg/g	± 0.32	19.29	µg/g	0.83	100.1	4.28	22
α-Tocopherol	1869, Infant Formula	217.2	µg/g	± 6.2	215.51	µg/g	9.58	99.2	4.44	4
	3290, Dry Cat Food	602	µg/g	± 55	606.67	µg/g	10.23	100.8	1.69	8
Vitamin D_3_	1869, Infant Formula	12.93	µg/100 g	± 0.31	13.07	µg/100 g	0.26	101.1	1.97	3
	1546a, Meat Homogenate	0.256	µg/100 g	± 0.053	0.22	µg/100g	0.01	86.9	2.29	7
Vitamin D_2_	1869, Infant Formula	14.02	µg/100 g	± 0.73	13.7	µg/100 g	0.50	97.7	3.65	4
Cholesterol	1869, Infant Formula	13.02	mg/100 g	± 0.47	13.16	mg/100 g	0.25	101.0	—^c^	2
	1546a, Meat Homogenate	71.7	mg/100 g	± 2.2	74.33	mg/100 g	2.53	103.7	3.41	7

a U_95%_ = 95% uncertainty confidence.

b Retinol, all-*trans* + 13-*cis* isomers.

c — = Not applicable.

### Matrix Spikes

Matrix spikes of 1×, 2×, and 5× were investigated on the automated method on infant formula, gummy vitamins, and salmon jerky. The automated method produced recoveries for retinol, α-tocopherol, cholecalciferol, and cholesterol between 90 and 110% (Table 2). Infant formula represents the low-content matrix, while gummy vitamins and salmon jerky represent the high-content test materials. No matrix interferences were observed from any of the matrixes tested.

**Table 2.** Matrix spike recovery of vitamins A, E, D, and cholesterol from infant formula, gummy vitamins, and salmon jerky

Matrix	Retinol^a^ (vitamin A)				α-Tocopherol (vitamin E)				Cholecalciferol (vitamin D)				Cholesterol			
	Recovery, μg	SD, μg	*n*	Spike recovery, %	Recovery, μg	SD, μg	*n*	Spike recovery, %	Recovery, μg	SD, μg	*n*	Spike recovery, %	Recovery, μg	SD, μg	*n*	Spike recovery, %
Infant formula (IF)	7.1	0.05	8		234.2	3.12	8		9.0	0.33	10		15.1	0.60	12	
IF + spike ×1	14.6	0.27	4	109.1	445.1	7.90	4	108.8	16.2	1.36	3	98.7	27.5	1.20	3	91.7
IF + spike ×2	22.1	0.31	4	108.3	649.9	9.99	4	107.5	24.6	1.39	3	96.7	61.8	1.40	3	96.0
IF + spike ×5	43.0	1.92	4	103.9	1265.2	36.50	4	106.9	54.3	2.59	3	107.6	96.0	2.65	3	95.0
Gummy vitamins (GV)	79.7	1.11	8		3635.4	62.31	8		—^b^	—	—	—	—	—	—	—
GV + spike ×1	156.5	1.41	4	100.0	7037.3	157.33	4	108.9	—	—	—	—	—	—	—	—
GV + spike ×2	229.0	0.30	4	97.5	9809.1	41.57	4	99.1	—	—	—	—	—	—	—	—
GV + spike ×5	457.7	1.33	4	99.2	19411.6	132.61	4	101.8	—	—	—	—	—	—	—	—
Salmon jerky (SJ)	—	—	—	—	—	—	—	—	30.3	1.07	13		76.3	2.42	10	
SJ + spike ×1	—	—	—	—	—	—	—	—	58.2	1.51	3	92.7	152.9	3.69	3	102.7
SJ + spike ×2	—	—	—	—	—	—	—	—	84.0	0.94	3	90.7	210.9	3.97	3	91.7
SJ + spike ×5	—	—	—	—	—	—	—	—	170.1	3.62	3	93.3	489.6	2.26	3	110.3

a Retinol, all-*trans* + 13-*cis* isomers.

b — = Not applicable.

### Method Comparison of Food and Pet Food

**Figure 1.**
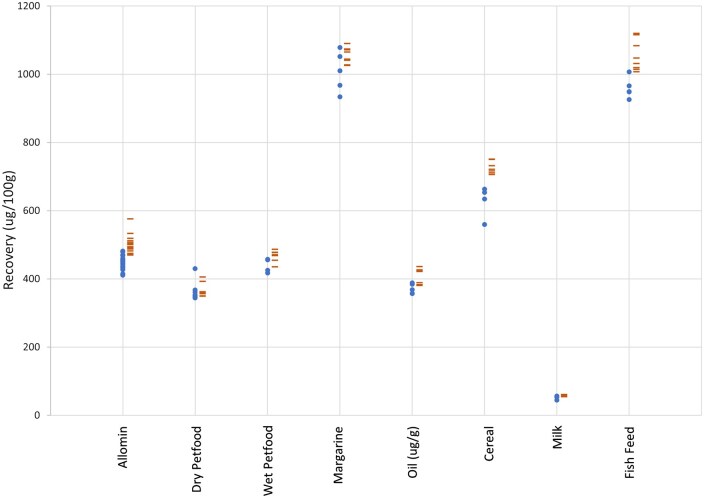
Retinol from food and pet food (•, EN method; -, automated method). Method correlation, R, 0.9958 (excluding oil).

**Figure 2.**
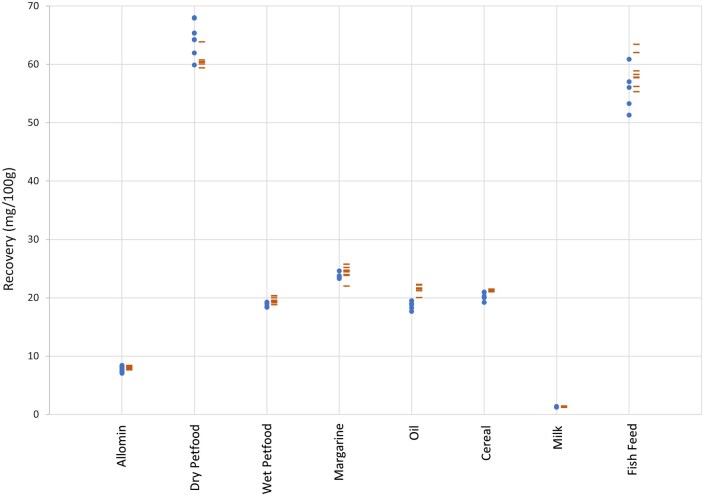
α-Tocopherol from food and pet food (•, EN method; -, automated method). Method correlation, R, 0.9922.

**Figure 3.**
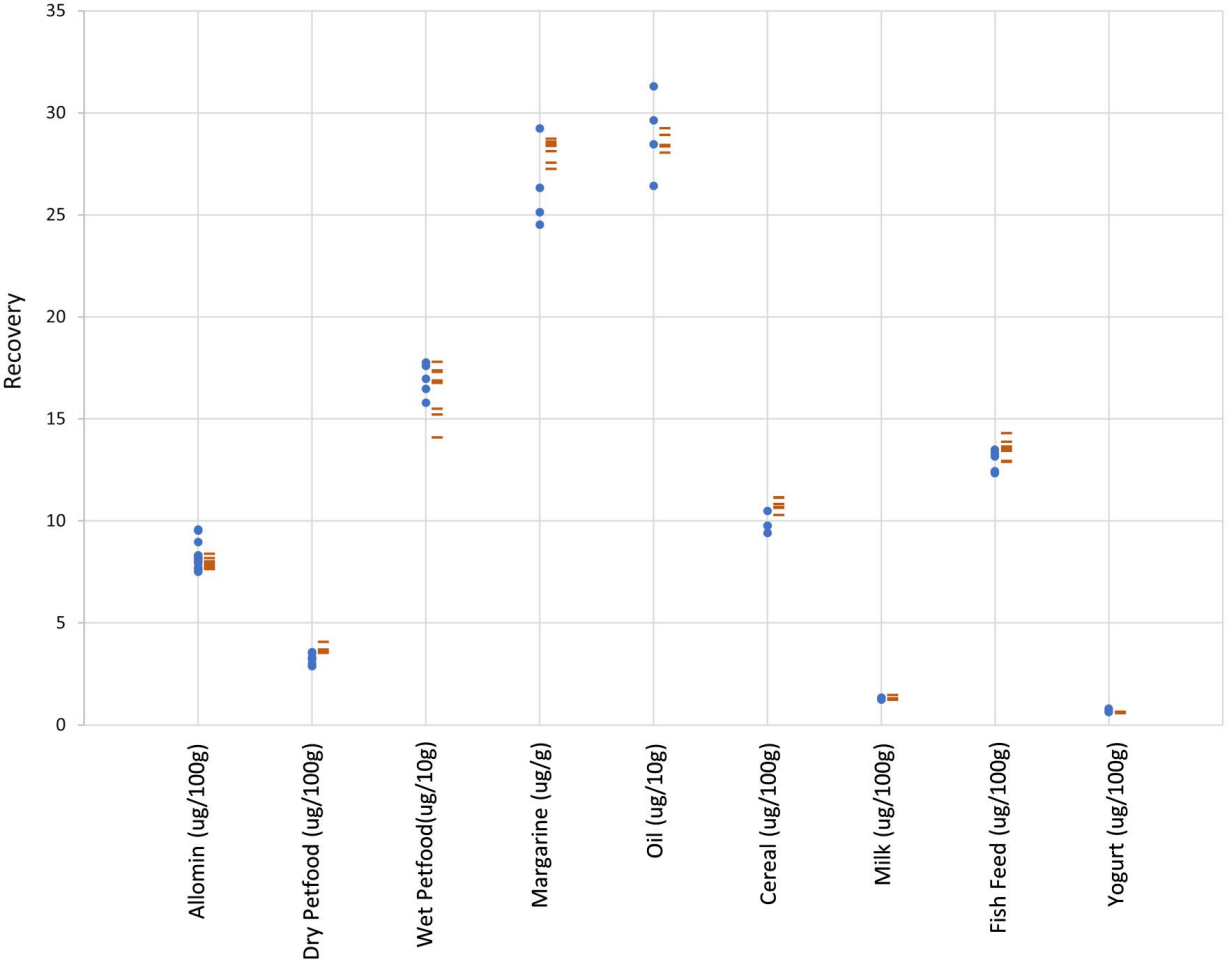
Cholecalciferol from food and pet food (•, EN method; -, automated method). Method correlation, R, 0.9998 (excl. margarine).

The manual methods (EN 12823–1, EN12822, and EN 12821) used to compare against the automated method employ ambient overnight saponification and liquid–liquid extraction while the automated method saponified at 75°C for 45 min and extracted with SPE. Quantification of analytes was performed in the same location, on the same HPLC system. Food and pet food test materials included a wide range of fat (1–80%), protein (0–40%), and carbohydrate (0–46%) content. Retinol results were significantly higher with the automated method than the manual method in five out of eight test materials (infant formula, wet pet food, oil, cereal, and fish feed), while the other three test materials showed no significant difference (dry pet food, margarine, and milk; Table 3 and Figure 1). α-Tocopherol results on the automated method were higher in three out of eight test materials (wet pet food, oil, and cereal), lower in only one test material (dry pet food), and not different in four out of eight test materials (infant formula, margarine, milk, and fish feed; Table 3 and Figure 2). Vitamin D_3_ results were higher with the automated method in three out of nine test materials (dry pet food, cereal, and fish feed), while all other test materials showed no difference between methods (Table 3 and Figure 3).

**Table 3.** Vitamins A, E, and D from food and pet food by EN and automated methods

		EN method				Automated method			
**Analyte**	**Test material**	**Mean, μg/100 g**	**SD, μg/100 g**	**RSD, %**	***n***	**Mean, μg/100 g**	**SD, μg/100 g**	**RSD, %**	***n***

Retinol	Infant Formula	448	21.2	4.7	20	501^a^	22.6	4.5	24
	Dry Petfood	363	28.4	7.8	8	369	21.9	5.9	7
	Wet Petfood	439	20.7	4.7	4	468^a^	16.0	3.4	8
	Margarine	1009	59.1	5.9	5	1055	23.5	2.2	8
	Oil	37119	1498	4.0	5	40688^a^	2289	5.6	8
	Cereal	628	46.7	7.4	4	727^a^	18.1	2.5	7
	Milk	51.6	6.13	11.9	3	58.4	2.10	3.6	7
	Fish Feed	959	30.4	3.2	5	1056^a^	45.3	4.3	8

	**Test material**	**Mean, mg/100 g**	**SD, mg/100 g**	**RSD, %**	***n***	**Mean, mg/100 g**	**SD, mg/100 g**	**RSD, %**	***n***

α-Tocopherol	Infant Formula	7.89	0.40	5.0	20	7.89	0.20	2.1	24
	Dry Petfood	64.6	2.8	4.3	8	60.8^a^	1.4	2.4	7
	Wet Petfood	18.8	0.41	2.2	4	19.6^a^	0.58	2.9	8
	Margarine	23.8	0.50	2.1	5	24.3	1.1	4.6	8
	Oil	18.7	0.72	3.8	5	21.7^a^	0.78	3.6	8
	Cereal	20.3	0.73	3.6	5	21.2^a^	0.20	0.7	7
	Milk	1.30	0.07	5.2	3	1.30	0.08	6.3	7
	Fish Feed	55.7	3.7	6.6	5	58.7	2.8	4.7	8

	**Test material**	**Mean, μg/100 g**	**SD, μg/100 g**	**RSD, %**	***n***	**Mean, μg/100 g**	**SD, μg/100 g**	**RSD, %**	***n***

Cholecalciferol	Infant Formula	8.24	0.55	6.7	20	9.78	0.2	2.1	24
	Dry Petfood	3.21	0.26	8.1	8	3.75^a^	0.3	5.3	6
	Wet Petfood	169	8.09	4.8	5	164	12.9	7.9	8
	Margarine	2630	209	7.9	4	2821	53.5	1.9	8
	Oil	290	20.5	7.1	4	286	4.7	1.7	5
	Cereal	9.86	0.46	4.6	4	10.9^a^	0.33	3.0	7
	Milk	1.30	0.05	3.6	4	1.27	0.06	5.1	7
	Yogurt	0.710	0.08	11.9	3	0.630	0.0	4.7	8
	Fish Feed	12.9	0.49	3.8	6	13.5^a^	0.46	3.4	8

a *P* ≤ 0.05.

**Table 4.** Vitamins A and E from AAFCO feeds by EN and automated methods^a^

Analyte	Test material	AAFCO No.	AAFCO Proficiency Program				Automated method			
			Mean, µg/g	SD, µg/g	RSD, %	*n*	Mean, µg/g	SD, µg/g	RSD, %	*n*
Retinol	Equine Feed	202121	6.97	1.1	15.7	14	7.14	1.0	14.6	5
	Beef Feed	202123	3.89	0.94	24.1	15	3.55	0.19	5.4	4
	Pig Feed	202124	0.660	0.19	28.6	10	0.49	0.14	29.6	4
	Dry Dog Feed	202125	5.05	1.2	24.1	16	6.67^b^	0.69	10.3	15
	Poultry Feed	202126	8.22	0.88	10.7	5	9.44	1.5	16.0	4
	Rabbit Feed	202127	2.74	0.60	21.7	11	2.73	0.67	24.6	4
	Sheep Feed	202128	15.3	3.0	19.3	14	16.8	2.6	15.6	4
	Goat Mineral	202199	132	19	14.0	14	130	9.2	7.0	4
	Hog Finisher	202130	1.44	0.83	57.5	6	0.31^b^	0.19	62.0	4
	Beef Feed	202131	12.5	3.1	25.0	10	11.8	3.6	30.4	4
	Poultry Feed	202132	2.57	0.32	12.5	6	2.64	0.08	3.0	4
α-Tocopherol	Equine Feed	202121	397	55	13.7	11	452.56	28	6.3	5
	Beef Feed	202123	83.3	9.3	11.2	14	88.51	6.2	7.1	4
	Pig Feed	202124	44.3	7.2	16.2	12	40.78	0.87	2.1	4
	Dry Dog Feed	202125	161	49	30.3	16	234.34^b^	7.9	3.4	15
	Poultry Feed	202126	56.3	15	25.8	7	54.07	0.90	1.7	4
	Rabbit Feed	202127	89.9	21	23.9	8	85.39	2.2	2.5	4
	Sheep Feed	202128	209	32	15.1	19	208.49	3.0	1.4	4
	Goat Mineral	202199	1093	211	19.3	11	1069.3	44	4.1	4
	Hog Finisher	202130	158	24	15.3	8	257.84^b^	11	4.2	4
	Beef Feed	202131	117	23	19.7	9	133.43	3.9	2.9	4
	Poultry Feed	202132	43.9	4.2	9.6	6	34.59^b^	0.47	1.4	4

a Method correlation: Retinol, *R*, 0.9753 (excluding Goat Mineral); α-Tocopherol, *R*, 0.9367 (excluding Goat Mineral).

b *P* ≤ 0.05.

A high correlation was seen between the automated and manual methods when comparing retinol (*R*, 0.996), α-tocopherol (*R*, 0.992), and vitamin D_3_ (*R*, 0.999) over nine different foods and pet foods. There was no bias (intercept), retinol, 3.3 µg/100 g; α-tocopherol, 0.97 mg/100 g; and vitamin D_3_, 0.51 µg/100 g, and no distortion (slope), retinol, 1.08; α-tocopherol, 0.98; and vitamin D_3_, 0.98, over the range of test materials.

**Figure 4.**
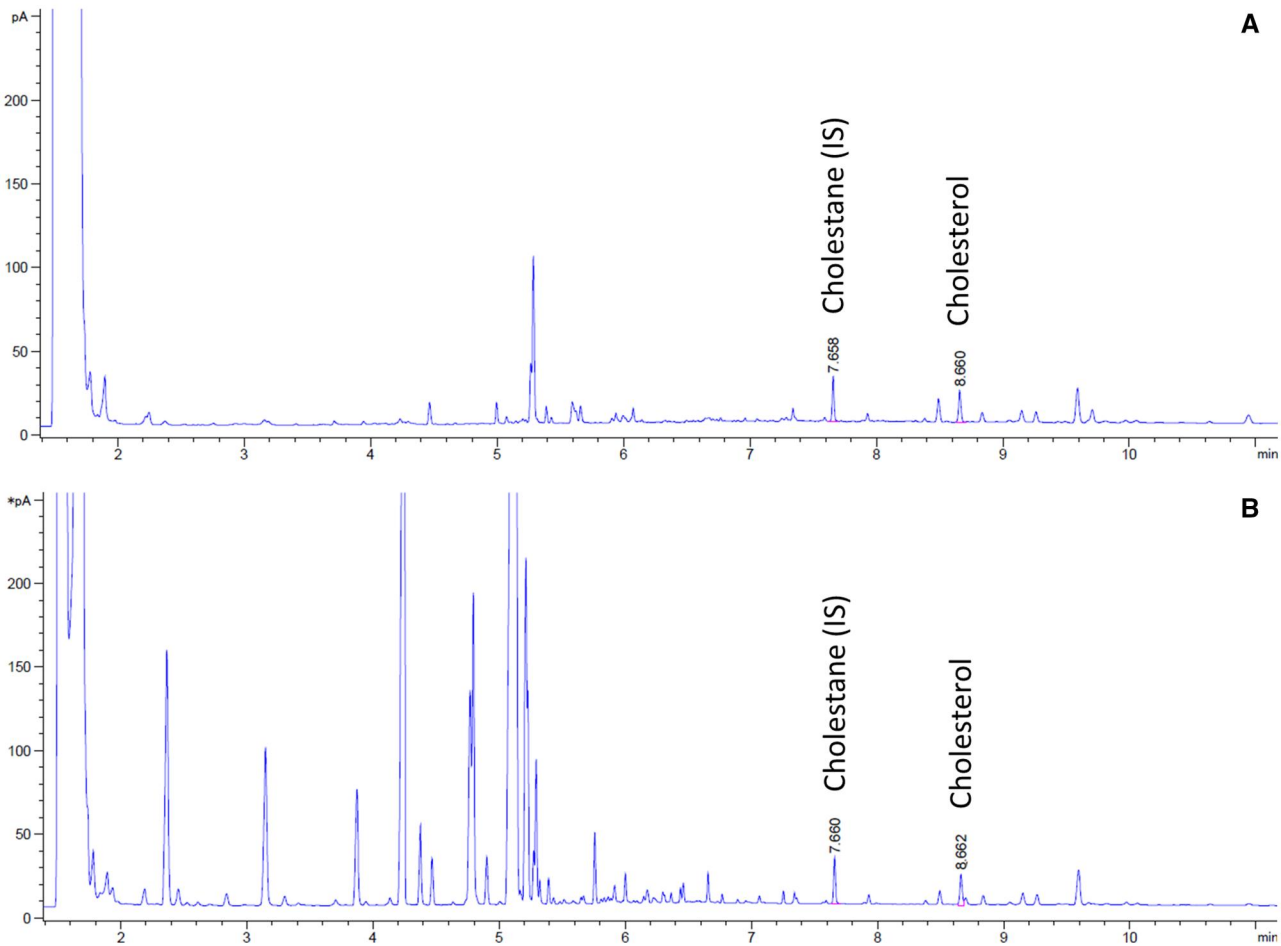
GC–FID chromatograms. Cholesterol analysis from infant formula: (A) automated method; (B) liquid–liquid extraction.

Chromatograms for cholesterol analysis indicated better cleanup from SPE than from liquid–liquid extraction. Figure 4 shows chromatograms from both methods with infant formula as the matrix. Although both methods yield good peak separation and similar results (results not shown), SPE showed fewer background peaks than liquid–liquid extraction.

### Animal Feed Proficiency Test Materials

Retinol and α-tocopherol recovery from animal feeds by the automated method were compared against recoveries from the AAFCO proficiency test materials. The AAFCO group consisted of approximately 20 participating laboratories using a variety of method references but mainly AOAC Method **974.29** (retinol) and AOAC Methods **948.26** and **971.30** (α-tocopherol). Results from the automated method correlated well with average retinol and α-tocopherol results from the AAFCO group. Retinol correlation was R, 0.975 (when excluding high-content, Goat Mineral), while α-tocopherol correlation was R, 0.937 (when excluding high-content, Goat Mineral). Goat Mineral retinol content is about 10 times that of the other feeds, so it was excluded in correlative statistical analysis so as to not skew results. Typically, feed analysis requires a 10–40 g test portion because retinol and vitamin D_3_ are not always dispersed well within the test material matrix. For this data set, the automated method used 5 g feed test portions. The FLEX instrument also can run methods that (1) analyze larger test portion sizes with addition of a filter aid and (2) analyze aliquots of enzyme-treated homogenized slurried test portions.

Detection and quantitation limits (LOD and LOQ) were calculated with HPLC–DAD/FLD as quantitation instrumentation. Retinol was quantified with an external calibration curve in a linear range of 0.046–41 µg/mL. The LOQ was 0.046 µg/mL when no test material matrix was present. α-Tocopherol was also quantified with an external calibration curve, in a linear range of 1.0–315 µg/mL. The LOQ was 4.1 µg/mL when no test material matrix was present.

The automated method has been extensively tested on various test materials, against various manual methods, and verified by laboratories who use the automated method. Test materials included all nine sections of the AOAC food triangle, spanning complex food formulations to high-protein, high-fat, and high-carbohydrate content.

## Discussion

The automated method for isolation and purification of fat-soluble vitamins proved to be very effective. The instrument was designed to take certain key factors into consideration. For example:

- Nitrogen is used to purge the system and to control filtration and elution rate in the system. At the beginning of each analysis, each chamber of the instrument is purged with nitrogen, to produce an inert environment which serves to protect sensitive analytes from oxidation.
- Digestion vessels were designed to hold the test portion, allow for magnetically coupled mixing, filtration, and the quantitative transfer of the test solution to the SPE columns. This was achieved by having a hydrophilic filter in the bottom of the vessel and then having an outlet in the bottom of the vessel. The selective filter serves as an impervious layer which is able to hold aqueous solutions without loss. The vessel can therefore be placed on a balance and the test portion can be weighed directly into it. With the addition of ethanol during saponification, the surface tension of the hydrophobic filter is reduced, allowing passage of the liquid. After saponification is completed, the vessel contents are filtered and transferred to the SPE columns by nitrogen pressure. Digestion vessels have a side port, which allows for manual additions of internal standards. The two-part vessel design also allows for inclusion of lofted filter types which aid in filtration of high-fiber test materials.
- The gang valve between the digestion vessels and SPE columns was designed to have no hold-up space.
- SPE columns are designed to retain approximately 70 mL of a polar solution, which can accommodate analysis of large test portion sizes.
- Evaporation is optimized using a nitrogen flow rate to agitate, disperse, and exhaust solvent from the temperature-controlled recovery vessels. This design evaporates solvent from four vessels at the same time in an inert atmosphere, which is an improvement on the rotary evaporator, which contains a heat source, a vacuum source, and a rotation station, for evaporation of one test portion at a time.
- The FLEX instrument incorporates numerous pressure, temperature, and liquid sensors to enable hands-free operation. After the addition of the test portion there is no manual intervention required by the operator to complete the analysis.

Saponification can be accomplished in a variety of conditions, from room temperature for 16 h to heated saponification (70–100°C) for 15–45 min (2, 5–13). The automated method employs heated saponification (75°C) for 45 min. However, saponification time and temperature are adjustable to allow for the range of conditions described in the methods above.

The extraction process in manual methods includes liquid–liquid extraction (7, 10–13) and SPE (2–3, 5, 14–15). They are used to separate fat-soluble vitamins and analytes from the polar chemicals and compounds in the saponified solution. Methods that employ SPE technology typically only extract a portion of the saponified solution (2–5, 16), or they use SPE to purify supernatant obtained after centrifugation (17). The automated method extracts the whole saponified test portion on high-capacity SPE columns.

The polarity of an analyte, sorbent, and solvent play an important role in the rate of elution. In the automated method, with hexane as solvent and a silicate-based sorbent, beta-carotene and α-tocopherol elute first, while cholecalciferol, β-tocopherol, and retinol elute next. γ-Tocopherol and δ-tocopherol are too polar to fully elute with hexane. By increasing solvent polarity, e.g., 5% THF in hexane, γ-tocopherol will fully elute, but δ-tocopherol will only partially elute.

Three variables dictate the test portion size required for fat-soluble vitamin analysis: (1) analyte content, (2) homogeneity of the analyte within the test material matrix, and (3) sensitivity of the quantitation device. Retinol and vitamin D are often found at low levels (e.g., milk contains 55 µg/100 g retinol and 1.3 µg/100 g vitamin D) and require larger test portions for analysis. Food and feed also can be fortified with stabilized encapsulated vitamins. Encapsulation often consists of coating with cross-linked gelatin (18) to form prilled beadlets. These high-concentration beadlets limit the dispersion in the test matrix. To improve the precision of the analysis, the test portion of fortified test materials must be increased. Some researchers have attempted to disperse encapsulated additions through grinding of the test material but still found poor retinol precision in a 10 g test portion (10.5–24.7% RSD) when compared to 100 g test portions (2.26–10.7% RSD; 17). USP 2040 and 711 (19, 20) recommend using proteolytic enzymes, pepsin or pancreatin, to disperse and release vitamin A. Gray et al. (21) proposed a revision to USP 2040 and 711 by including proteolytic enzymes papain or bromelain. We have found that retinol precision improved with (1) increasing test portion size and (2) bromelain was effective in dispersing retinol within a test portion slurry, increasing precision. Homogenization was achieved by digesting a 40 g feed slurry with bromelain for 60 min. Subsequently, only a small aliquot of the feed slurry (containing a 5 g test portion) was saponified and extracted with the FLEX method. Retinol precision was significantly reduced to <10%.

The type of quantitation device influences the LOQ, which inadvertently influences the test portion size requirement. Quantitation by MS is highly selective and sensitive, compared to UV-Vis, DAD, or FL detection and therefore can accommodate test materials with lower analyte concentration. In most cases, standard detection devices are sufficient, but if greater sensitivity is required, MS is essential.

Low analyte content, poorly dispersed analyte, encapsulation, and less sensitive quantitation instrumentation all will drive the need for larger test portions (5–20 g) than are typically used in proximal analysis (0.5–2 g; 22). The automated method generally allows for up to 10 g of solid test portions and 25 g of liquid test portions.

The FLEX instrument was designed to include custom functionality. Digestion, filtration, extraction, and evaporation are processes frequently used in an analytical laboratory. This unique design element makes this instrument a valuable tool for method development and includes the following:

- Method functions are modular and can be strung together in a sequential way to form an automated custom method.
- Digestion vessel filters are available in a variety of different lofts, porosities, and compositions.
- The three-way gang valve allows for drainage of solutions from the digestion vessels to the SPE columns or to waste. This allows for selective extraction.
- Empty SPE cartridges are available for experimentation with alternative sorbent types.

Numerous custom methods have been created to automate other troublesome wet chemistry methods. One such example is the extraction of esterified vitamins with a mixture of DMSO and hexane from high-content supplements. This method involves heated liquid–liquid extraction (DMSO/hexane) of vitamin premixes. In this case, the immiscible solvents are mixed vigorously, at elevated temperatures, in the digestion vessels. After a set amount of time, extraction is completed, and the solution is drained onto the SPE columns, where DMSO is retained by the column and the analyte is eluted with hexane. By automating this method, heated liquid–liquid extraction could be accomplished, hands-free.

## Conclusions

An automated method using a FLEX instrument for the isolation and purification of fat-soluble vitamins and cholesterol for chromatographic analysis was validated. Four key micronutrients (retinol, α-tocopherol, vitamin D_3_, and cholesterol) were accurately recovered from reference materials, various food and pet food matrixes, and animal feeds with minimum technician involvement. The automated method has the added benefits of automation of a multistep analysis, reducing solvent usage per assay, limiting technician exposure to harmful chemicals, limiting injuries from shaking separatory funnels, and reducing labor cost. Custom software streamlines method development by controlling, monitoring, and logging digestion, extraction, and evaporation parameters. Additionally, precision is improved by combining three sequential method steps into one closed system.

## CRediT Author Statement

Marleen van Aardt (Conceptualization [Equal], Data curation [Lead], Formal analysis [Lead], Investigation [Lead], Methodology [Lead], Project administration [Lead], Validation [Equal]), Andrew R Komarek (Conceptualization [Equal], Methodology [Equal]), Michael Roche (Formal analysis [Supporting]), and Elise Ivarsen (Formal analysis [Supporting])
